# Brazilian Protocol for Sexually Transmitted infections 2020: approaching sexually active individuals

**DOI:** 10.1590/0037-8682-628-2020

**Published:** 2021-05-17

**Authors:** Maria Alix Leite Araujo, Juliana Uesono, Nádia Maria da Silva Machado, Valdir Monteiro Pinto, Eliana Amaral

**Affiliations:** 1 Universidade de Fortaleza (UNIFOR), Programa de Pós-Graduação em Saúde Coletiva, Fortaleza, CE, Brasil; 2 Ministério da Saúde, Secretaria de Vigilância em Saúde, Brasília, DF, Brasil; 3 Secretaria do Estado da Saúde de São Paulo, CRT-IST/Aids, Programa Estadual de IST/Aids, São Paulo, SP, Brasil.; 4 Secretaria Municipal da Saúde, Coordenação Municipal de IST/Aids, São Paulo, SP. Brasil.; 5 Universidade Estadual de Campinas, Faculdade de Ciências Médicas, Campinas, SP, Brasil

**Keywords:** Sexuality, Sexually transmitted infections, Disease prevention, Diagnosis screening programs, Clinical protocols, Surveillance

## Abstract

This article aims to present concepts and clinical practices recommended to approach people with active sex life. These concepts are an integral part of the recommendations of the Clinical Protocol and Therapeutic Guidelines for Comprehensive Care for People with Sexually Transmitted Infections (STI), published by the Ministry of Health of Brazil in 2020. The article proposes a comprehensive approach to sexuality for health promotion. It presents significant aspects of the communication process that must develop, without prejudice and judgment, focusing on sexual and reproductive health. It also highlights relevant points about the exercise of sexuality at specific stages of life, recommending assessment of risks and vulnerabilities and screening for STI and condom use. In this way, it is possible to contribute to exercise their sexuality fully, responsibly, and safely.

## Highlighted excerpt

Sexual health is the physical, emotional, mental, and social wellbeing associated with the exercise of sexuality, with sexual and reproductive rights considered fundamental.

## FOREWORD

This article aims at updating the chapter on Sexual Health: an approach centered on sexually active individuals of the Clinical Protocol and Therapeutical Guidelines (PDCT) for Comprehensive Care for People with Sexually Transmitted Infections (STI) 2020^1^. We highlight the main thematic points: the communication in approaching sexual health, sexuality in specific life stages, assessment of risks and vulnerabilities, STI tracing, and condom use. We made adaptations in the chapter items to make it more adequate regarding issues of other STIs different from HIV, as it has a specific clinical protocol^2^.

The PDCT was published by the Health Surveillance Department of the Brazilian Ministry of Health, based on official recommendations and discussions with experts. The National Committee approved it for Technology Incorporation to the Unified Health System (Conitec) in 2018^3^. It proposes a sensitive approach to sexuality, aiming at improving the health of sexually active individuals. 

## INTRODUCTION

Sexual and reproductive rights are considered fundamental, together with the rights to life, food, health, housing, and education for the complete exercise of citizenship^4^. The right of individuals and couples of all sexual orientations to have their sexual health preserved is recognized. Sexual health is defined as the physical, emotional, mental, and social wellbeing associated with the exercise of sexuality and not just the lack of sexual infections, disorders, or diseases^5^. It is considered an essential component for promoting human development^6^. It implies the exercise of safe and healthy sexual experiences without coercion, discrimination, or violence^7^. Finally, sex is understood as one of the essential dimensions of sexuality, not limited to genitality or reproduction^8^. 

## COMMUNICATION IN THE APPROACH TO SEXUAL HEALTH

The communication process plays a significant role in the improvement of the professional-patient relationship and, as a consequence, for the adherence to the recommendations and treatment^9^. Health professionals can be unprepared and feel embarrassed when it comes to approaching patients regarding STI and sexuality^10^. The offer of adequate training could minimize such embarrassment and contribute to qualifying such professionals' performance, aiming to make them familiar with the different concepts of gender, sexual orientation, and identity.

Health services must promote environments favorable to the dialog and embrace the different dimensions of the exercise of sexuality by sexually active individuals. We recommend a gradual approach, advancing from general aspects to the most specific ones^11^. Adequate approach to sexuality must encompass guidelines on prevention and identification of risk factors and vulnerabilities, sexual practices, and behaviors that favor STI contamination. 

To establish an association of trust, we need a clear and transparent approach, adequate to receptivity and the life context of the persons, which must be recognized as active individuals in the care process^10^. All services must favor the development of autonomy of the subjects for identifying solutions to their demands. The approach must take place free from prejudiced attitudes, labels, and stigmatization, understanding sexuality as part of the culture and the historical, social, and life context of each individual^4^. 

**FIGURE 1:**
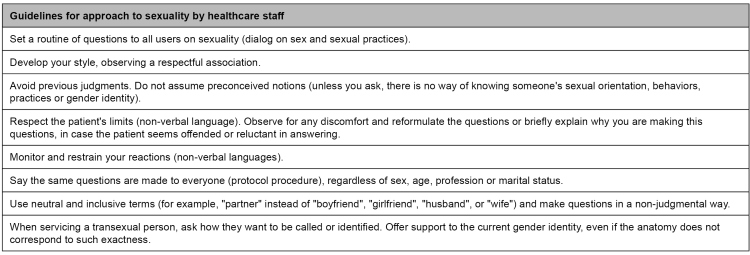
General guidelines for approach to sexuality by healthcare staff.

We recommend to the professional to assure that the person is comfortable to talk about such themes, informing them that those are questions made routinely in the healthcare service, regardless of sex, sexual orientation, age, professional activity, and marital status. Emphasis should be placed on the secrecy and confidentiality of information^12^ ^,^ ^13^ General guidelines on communication can be found in Figure 1.

Telemedicine has currently been gaining ground in disclosing information, promotion and prevention, expanding the scope of healthcare services, especially in a continental country with regions that are hard to reach. Useful communication techniques in servicing people with STI are also needed in telemedicine^14^. For this reason, it is necessary to incorporate its use in the service to people with STI, respecting the ethical limits and current recommendations regarding data storage, handling and transmission, as well as confidentiality, privacy and the guarantee of professional secrecy^15^.

## SEXUALITY IN SPECIFIC LIFE STAGES

Adolescence is a period of significant biological, psychological and social transformations. Physical alterations, social interactions and the awakening of new interests reflect fast and profound changes that characterize this life stage. The way adolescents express and live sexuality is influenced by the quality of emotional and affective associations experienced with relevant people in childhood, integration with peers, transformation arising from growth and development, the start of reproductive capacity, beliefs, moral patterns, myths, and taboos, as well as the traditions of the family or society in which they are inserted^16^. 

In this stage, values, attitudes, habits, and behaviors are being formed, transformed, and consolidated, making adolescents more vulnerable, mainly because parents or guardians, school, and even health staff tend not to approach the aspects regarding the exercise of sexuality. Therefore, many times adolescents start their sexual lives without due orientation^17^.

Healthcare services can play a fundamental role, disclosing to adolescents information contributing to the awakening of a healthy sexual life and prevention of STI and unintended pregnancy. Such orientation must be based on dialog, allowing for autonomy and responsible attitudes^18^. The approach must comply with confidentiality and privacy principles, indispensable for trust and respect between adolescents and health professionals^19^. It also must take place from the point of view of comprehensive care, providing access to different technologies associated with combined prevention^16^.

During gestation, sexual relations do not pose a risk to pregnancy, except in unique obstetric situations (membrane rupture, cervical insufficiency, short cervix, or premature delivery). However, we should not ignore the possibility of a pregnant woman getting an STI that harms the gestation's prognosis, or vertically transmitting the disease. For this reason, healthcare staff must approach routine questions associated with the sexual health of pregnant women and their sexual partners and offer HIV, syphilis, and hepatitis B and C tests in prenatal care^18^. 

Older adults presented an increase in the number of HIV and syphilis cases over the last years, drawing attention to the role of sexuality in this age group^20^ ^,^ ^21^. There are essential aspects that increase vulnerability, as lower genital lubrication in women, and male erection difficulty^20^ ^-^ ^22^. Besides, it is a generation that was not sexually initiated with safe sex. 

## RISK ASSESSMENT, VULNERABILITIES, AND STI TRACING

**FIGURE 2:**
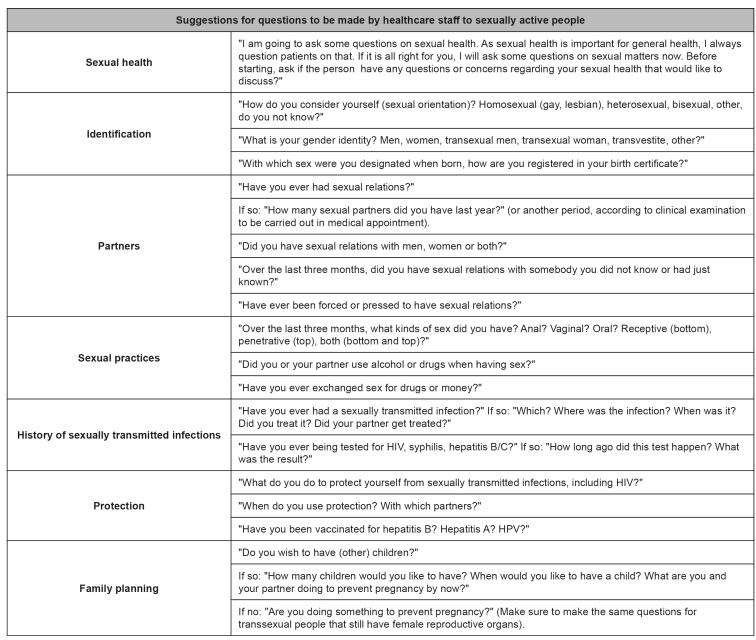
Routine service questions for assessment of the risk of sexually transmitted infections**.**

**Source:** adapted from Rocha et al., 2019^10^; Nusbaum, Hamilton, 2002^30^; Workowski, Bolan, 2015^31^.

In risk assessment for STI in sexually active individuals, we recommend investigating structured questions, identifying factors associated with sexual practices and behaviors, and alcohol and drug use. From the contents obtained, it is possible to make an adequate assessment for risk management and identify opportunities for recommending preventive actions. The professional must individually provide the attendance, and in a private environment^1^ ^,^ ^16^ ^,^ ^23^. Figure 2 presents questions for the attendance towards risk assessment.

Risk assessment can guide STI tracing. After identifying clinical cases, it is crucial to call and treat the sexual partnerships, aiming to interrupt the chain of infection, prevent complications, and avoid reinfections^1^. In Brazil, the PDCT recommends screening asymptomatic subgroups^1^ to identify and treat infected individuals earlier, looking to prevent STI dissemination and their complications^24^. 

European guidelines recommend managing sexual partners of people with STI, indicating emotional support and contact identification and notification, through a strategy guided by pattern operational procedure for control, monitoring, treatment, and report of cases^25^. 

**FIGURE 3:**
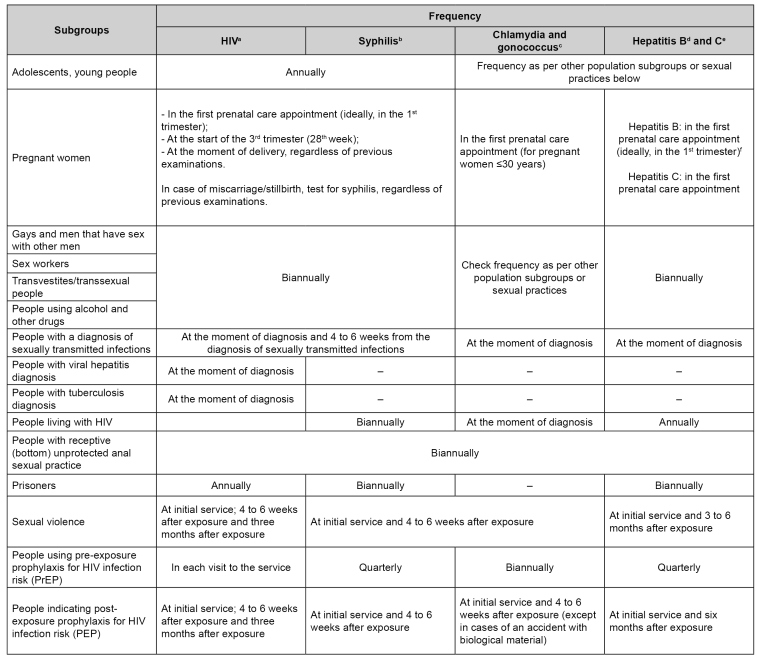
Tracing indication for sexually transmitted infections according to population subgroups.

Brazil presents a trend of HIV and syphilis increase in the population between 13 and 29 years of age^20^ ^,^ ^21^. For this reason, it recommends the annual tracing of such infections in people up to 30 years old who are sexually active. Other diseases are screened depending on population groups and sexual practices that expose people to more significant risks. Figure 3 presents recommendations for tracing people in any age group. 

a) HIV: preferably with a rapid test; b) Syphilis: preferably with a rapid test; c) Chlamydia and gonococcus: chlamydia and gonococcal detection through molecular biology. Research according to sexual practice: urine (urethral), endocervix samples, genital secretion. For extragenital samples (anal and pharyngeal ones), use tests to validate such anatomic collection sites; d) Hepatitis B: preferably with a rapid test. We recommend vaccinating everyone susceptible to hepatitis B. Susceptible person is the one who does not have a record of a complete vaccine scheme and presents non-reacting surface antigen for hepatitis B virus (or non-reacting rapid test for hepatitis B); e) Hepatitis C: preferably with a rapid test; f) If the pregnant woman has not carried out tracing in prenatal care, perform a rapid test for hepatitis B at the moment of delivery. Hepatitis B vaccine is safe during pregnancy, and susceptible women must be vaccinated.

It is important to trace some infections, such as chlamydia and gonococcus, aiming at preventing pelvic inflammatory disease that, even if asymptomatic, can reduce chances of pregnancy^26^. In Brazil, screening for chlamydia infections is recommended for pregnant women younger than 30 years old due to the high prevalence of infection in this age group^27^. 

Figure 3 presents recommendations for tracing STI according to population subgroups, as proposed by the PDCT for Comprehensive Care for People with STI 2020^1^.

## CONDOM USE

Using female, or male condoms is a preventive strategy that should be offered to sexually active people, in order to reduce the risk of HIV and other STI transmission and prevent pregnancy^28^. Despite the low adherence and acceptance of the female condom, it is deemed necessary in situations where it is difficult for the women to negotiate the use of the male condom^29^.

**FIGURE 4:**
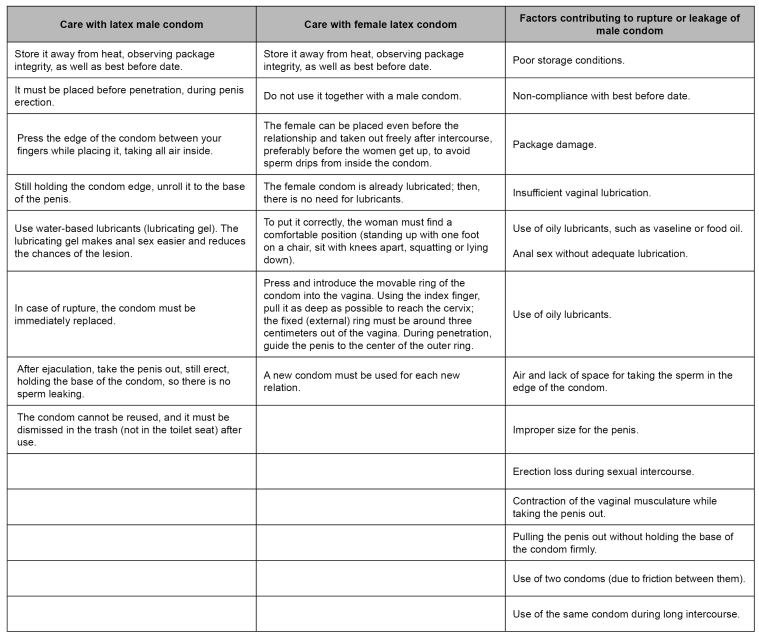
Use and adequate conservation of male and female condom.

The offer of condoms must take place without the quantity restrictions and without requiring identification documents. Figure 4 presents the guidelines for conservation and correct use of male and female condoms. They should be part of the healthcare staff's approach in all services, especially highly vulnerable individuals.

## CONCLUDING REMARKS

Healthcare professionals must incorporate sexuality into services to sexually active individuals, mainly those with STI complaints. This approach must develop, without prejudice and judgment, with a focus on sexual and reproductive health. In this way, it is possible to contribute to exercise their sexuality fully, responsibly, and safely. The right preventive approach can favor the decrease of STIs and their consequences.
